# Selenoprotein P Modulates Methamphetamine Enhancement of Vesicular Dopamine Release in Mouse Nucleus Accumbens Via Dopamine D2 Receptors

**DOI:** 10.3389/fnins.2021.631825

**Published:** 2021-04-13

**Authors:** Daniel J. Torres, Jordan T. Yorgason, Catherine C. Mitchell, Ayaka Hagiwara, Marilou A. Andres, Suguru Kurokawa, Scott C. Steffensen, Frederick P. Bellinger

**Affiliations:** ^1^Department of Cell and Molecular Biology, John A. Burns School of Medicine, University of Hawai‘i at Mânoa, Honolulu, HI, United States; ^2^Pacific Biosciences Research Center, University of Hawai‘i at Mânoa, Honolulu, HI, United States; ^3^Department of Physiology and Developmental Biology, Brigham Young University, Provo, UT, United States; ^4^Faculty of Pharmacy, Osaka Ohtani University, Osaka, Japan; ^5^Department of Psychology, Brigham Young University, Provo, UT, United States

**Keywords:** selenoprotein P, apolipoprotein E receptor 2, methamphetamine, dopamine, fast-scan cyclic voltammetry

## Abstract

Dopamine (DA) transmission plays a critical role in processing rewarding and pleasurable stimuli. Increased synaptic DA release in the nucleus accumbens (NAc) is a central component of the physiological effects of drugs of abuse. The essential trace element selenium mitigates methamphetamine-induced neurotoxicity. Selenium can also alter DA production and turnover. However, studies have not directly addressed the role of selenium in DA neurotransmission. Selenoprotein P (SELENOP1) requires selenium for synthesis and transports selenium to the brain, in addition to performing other functions. We investigated whether SELENOP1 directly impacts (1) DA signaling and (2) the dopaminergic response to methamphetamine. We used fast-scan cyclic voltammetry to investigate DA transmission and the response to methamphetamine in NAc slices from C57/BL6J SELENOP1 KO mice. Recordings from SELENOP1 KO mouse slices revealed reduced levels of evoked DA release and slower DA uptake rates. Methamphetamine caused a dramatic increase in vesicular DA release in SELENOP1 KO mice not observed in wild-type controls. This elevated response was attenuated by SELENOP1 application through a selenium-independent mechanism involving SELENOP1-apolipoprotein E receptor 2 (ApoER2) interaction to promote dopamine D2 receptor (D2R) function. In wild-type mice, increased vesicular DA release in response to methamphetamine was revealed by blocking D2R activation, indicating that the receptor suppresses the methamphetamine-induced vesicular increase. Our data provide evidence of a direct physiological role for SELENOP1 in the dopaminergic response to methamphetamine and suggest a signaling role for the protein in DA transmission.

## Introduction

The mesolimbic system facilitates the rewarding effects of stimuli such as food, social interaction, and drugs of abuse (Nestler and Carlezon, 2006). Central to this function is the release of the neurotransmitter dopamine (DA) in the nucleus accumbens (NAc) in the ventral striatum, from afferents originating in the midbrain ventral tegmental area. Mesolimbic DA transmission is an essential causative factor in addiction (Wise, 1998; Koob and Le Moal, 2001). Methamphetamine is an illicit and highly addictive psychostimulant that is a type of amphetamine, a class of drugs that potentiate dopaminergic transmission. Amphetamines inhibit DA uptake through the DA transporter (DAT), resulting in elevated levels of extracellular DA in the synapse (Seiden et al., 1993; Sulzer, 2011). They are also capable of entering DA terminals and inducing the release of DA from vesicles into the cytosol by disrupting vesicular monoamine transporter-2 (VMAT-2) function. The increased cytoplasmic DA results in reverse transport of DA through DAT, a phenomenon known as “DA efflux” (Hedges et al., 2018). Daberkow et al. (2013) reported that D-amphetamine also causes an increase in vesicular DA release, while other studies have disputed this finding (Siciliano et al., 2014). Excessive methamphetamine exposure is neurotoxic, primarily causing deterioration of dopaminergic terminals, and chronic use causes cognitive deficits (Seiden et al., 1988; Volkow et al., 2001; Johanson et al., 2006).

Selenium, an essential trace element, is required for proper brain function (Pillai et al., 2014). Proteins of the selenoprotein family incorporate the trace element to form selenocysteine (Sec), the 21st amino acid (Bellinger et al., 2009). Selenoproteins serve a variety of roles, most notably as antioxidants, and production is highly dependent on dietary selenium availability (Ogawa-Wong et al., 2016). Previous studies indicated an interaction between selenium and the DA system (Castano et al., 1993, 1995, 1997; Rasekh et al., 1997; Romero-Ramos et al., 2000). Selenium supplementation protects against methamphetamine-induced neurotoxicity in rodent and *in vitro* models (Imam et al., 1999; Kim et al., 1999), whereas selenium deficiency potentiates toxicity (Barayuga et al., 2013). Dietary selenium restriction lowers selenoprotein expression levels and can increase the turnover of DA and its metabolites in rodent striatum, as measured by *in vivo* microdialysis (Romero-Ramos et al., 2000). It is unclear, however, how selenium affects DA transmission and what function selenoproteins may have in DA release.

Selenoprotein P (SELENOP1) is a secreted glycoprotein produced primarily in the liver, and in lesser amounts in other tissue. SELENOP1 is unique among selenoproteins in that it contains 10 Sec residues instead of only one (Burk and Hill, 2009). SELENOP1 is primarily considered a selenium transporter that travels through the blood stream delivering selenium to different body regions including the brain. Genetic deletion of SELENOP1 decreases brain selenium content by roughly 50%, similar to the effects of long-term dietary selenium restriction (Nakayama et al., 2007). Dietary supplementation with excess selenium can restore brain selenium levels in SELENOP1 knockout (KO) mice through non-SELENOP1 mechanisms (Burk and Hill, 2015) and prevent most resulting neurological impairments (Hill et al., 2003; Schomburg et al., 2003; Nakayama et al., 2007). Therefore, we investigated SELENOP1 KO mice to determine how restricted selenium delivery to the brain influences dopaminergic transmission and responses to methamphetamine. We used fast-scan cyclic voltammetry (FSCV) to measure DA release and re-uptake events in mouse NAc brain slices (Yorgason et al., 2011).

This study provides the first evidence, to our knowledge, that a specific selenoprotein directly modulates DA transmission. Our findings demonstrate SELENOP1 signaling via apolipoprotein E receptor 2 (ApoER2) that is independent of selenium. This signaling limited DA release in the presence of methamphetamine, potentially contributing to the ability of selenium to protect against methamphetamine-induced neurotoxicity (Imam et al., 1999; Kim et al., 1999; Barayuga et al., 2013). Finally, our results complement previous reports that methamphetamine augments vesicular DA release in the striatum, a point of contention in amphetamine research (Daberkow et al., 2013; Siciliano et al., 2014).

## Materials and Methods

### Animals

All mouse care and experimental procedures were approved by the UH Manoa Institutional Animal Care and Use Committee (UH Manoa IACUC), protocol number 10–742, and conducted in accordance with the National Research Council’s *Guide for the Care and Use of Laboratory Animals* and the ARRIVE guidelines. We used SELENOP1 KO mice with a C57/BL6J background (Hill et al., 2003) initially obtained from Vanderbilt University, and WT C57/BL6J mice initially obtained from Jackson Laboratories. As homozygous male SELENOP1 KO mice are sterile (Hill et al., 2003), the strain was maintained by breeding with the C57/BL6J mice for breeders, and experiments used homozygous SELENOP1 KO offspring. When possible, homozygous WT littermates of SELENOP1 KO mice were utilized. All mice used were 3–5 months of age. Littermates were group-housed up to 5 in a cage on a light/dark cycle and allowed access to food and water *ad libitum*. Mice were fed standard lab chow (Envingo, Cat#2920X) containing 0.23 ppm selenium. For indicated experiments, SELENOP1 KO mice were supplemented with selenium by adding sodium selenite (1 mg/ml) to the drinking water following weaning. No other agents or conditions were utilized prior to tissue harvest for experiments. Studies utilized brain slices from both male and female mice. No significant or apparent sex differences were observed within wild-type (WT) or SELENOP1 KO groups in terms of basal measurements and methamphetamine response. Therefore, data from male and female mouse brain slices were combined within each comparison.

### Brain Slice Preparation

Brain slices containing NAc were obtained from WT and SELENOP1 KO mice and FSCV employed to assess DA release and reuptake at under baseline conditions and in the presence of methamphetamine. Methamphetamine and other pharmacological agents were applied to NAc slices via perfusion with artificial cerebral spinal fluid (ACSF) while monitoring changes in extracellular DA concentrations.

Mice were euthanized via rapid cervical dislocation to avoid effects of anesthetic remnants on neurophysiology. Mouse brains were removed and placed in ice-cold ACSF consisting of: 130.00 mM NaCl, 3.50 mM KCl, 10.00 mM glucose, 24.00 mM NaHCO_3_, 1.25 mM NaH_2_PO_4_, 1.50 mM MgSO_4_, 2.00 mM CaCl_2_, and bubbled with carbogen gas (95% O_2_/ 5% CO_2_). Coronal brain slices of 350 μm containing NAc were obtained using a Leica VT 1200 S vibrating blade microtome. Hemispheres of striatal slices were separated using a scalpel and placed into a slice incubation chamber containing oxygenated ACSF. Following recovery at room temperature for 30 min, slices were transferred to a heated water bath at 33°C for at least 30 min prior to experimentation.

### Voltammetric Recordings

**FIGURE 1:**
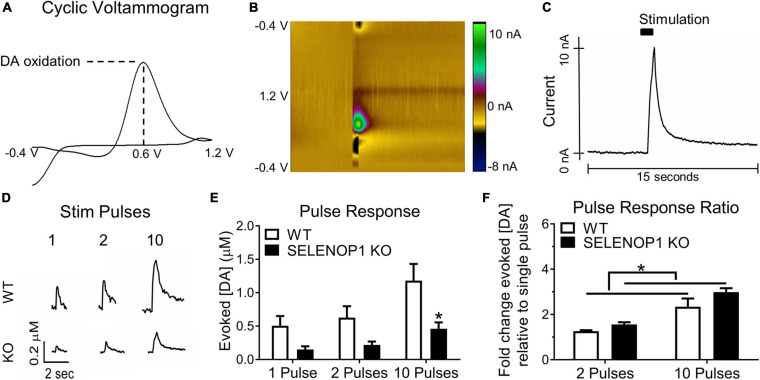
Deletion of SELENOP1 resulted in reduced evoked DA responses measured in NAc brain slices. **(A)** sample cyclic voltammogram using 10-pulse stimulation showing peak oxidation potential around 0.6 V and used to confirm DA detection. **(B)** 3-dimensional heat map depicting the measured current according to color scale (right) for each point in voltage sweep (*y*-axis), plotted over time (*x*-axis). **(C)** sample evoked DA signal derived from the peak current of the plot in **(B)**, following stimulation at 5 s. **(D)** representative traces from C57 WT mice and C57 SELENOP1 KO mice, aged 3–5 months, in response to 1-, 2-, and 10-pulse stimulation. **(E)** mean (± SEM) peak DA responses from 1-, 2, and 10-pulse stimulation. Two-way ANOVA revealed a significant effect of genotype on the amount of DA released (*F*_(1,24)_ = 17.38; *p* = 0.0003). A 1-pulse stimulation caused DA release from slices of WT mice (0.5 ± 0.15 μM; *n* = 5) and slices from SELENOP1 KO mice (0.15 ± 0.05 μM; *n* = 5), 2-pulse stimulation-induced DA release (0.63 ± 0.17 μM, *n* = 5 from WT mice compared to 0.22 ± 0.05 μM, *n* = 5 for SELENOP1 KO mice). A 10-pulse stimulation induced greater DA release in WT (1.2 ± 0.37 μM; *n* = 5) than in SELENOP1 KO mice (0.46 ± 0.12 μM; *n* = 5; *p = 0.0198, Tukey’s multiple comparisons test). **(F)** mean (± SEM) ratios of either 2-pulse-elicited responses or 10-pulse-elicited responses to 1-pulse-elicited responses. Two-way ANOVA revealed a significant effect of genotype (*F*_(1,16)_ = 4.962; *p = 0.0406). The ratio of 2-pulse to 1-pulse responses in SELENOP1 KO mice was 1.6 ± 0.1 μM; *n* = 5, and in WT mice it was 1.26 ± 0.04 μM; *n* = 5. The ratio of 10-pulse to 1-pulse responses was comparable between SELENOP1 KO and WT mice (2.9 ± 0.3 μM; *n* = 5 and 2.3 ± 0.1 μM; *n* = 5, respectively). All values reported are mean ± SEM.

For *ex vivo* FSCV experiments, brain slices were transferred to a slice recording chamber and constantly perfused with oxygenated ACSF at 33°C at a flow rate of 3 mL/minute. For recordings, a carbon fiber electrode (CFE) was placed ∼100 μm below the surface of the brain slice in the NAc shell under the guidance of a microscope with a 10× objective lens. The stimulating electrode was placed 100–200 μm from the tip of the CFE at the same depth of the CFE. Extracellular DA concentrations were measured using a Dagan CHEM-CLAMP voltage clamp amplifier. A command voltage (CV) was applied to the CFE and scanned linearly in a triangular waveform from −0.4 to 1.2 V at a rate of 400 V/second. The CV induces DA oxidation, resulting in a current conductance proportional to the concentration of extracellular DA present (Figure 1A).

For evoked DA release measurements, the CV was applied at a frequency of 10 Hz (every 0.1 s), and the resulting current response to each CV was measured to produce a cyclic voltammogram with a peak current response representing DA oxidation at its oxidation potential (∼0.6 V). A 1-min epochs were collected every 2 min coinciding with a single stimulation train. Cyclic voltammograms were regularly referenced to confirm the specificity of the current output to DA oxidation. Data were digitized using an NI-6221 analog-to-digital converter (National Instruments) and analyzed using Demon Voltammetry software (Yorgason et al., 2011). DA release was elicited via electrical stimulation, and the resulting signal analyzed to assess release and reuptake (Figures 1B,C). DA release was evoked using a 10-pulse train of 0.5 ms biphasic stimuli (370 μA) at 20 Hz every 2 min using an A365 Stimulus Isolator (World Precision Instruments) to simulate phasic DA release events (Ferris et al., 2013). In initial assessments, stimulation trains of 1-, 2-, and 10-pulses at 20 Hz were used to test the level of responsiveness to varying degrees of stimulation.

After observing 30 min of stable baseline responses (2-min epochs), methamphetamine in ACSF was applied via perfusion for 30 min, followed by washout with regular ACSF for another 30 min. In some experiments, chemicals were applied for at least 15 min prior to methamphetamine application and for total durations indicated in figures. Drugs and purified proteins were diluted in ACSF and delivered via perfusion during experiments. Methamphetamine was used at a working concentration of 10 μM (2 times the measured EC50 when applied to mouse NAc slices) (Hedges et al., 2018). Concentrations are indicated in the RESULTS sections for: Quinpirole (Sigma, Q102); Sulpiride (Sigma, S8010). Stock solutions were made up in Milli-Q water at a 10,000× concentration to minimize any potential effect on the osmolarity of ACSF chemical components.

### Electrode Fabrication and Calibration

Carbon fiber electrode were produced by inserting a 7 μm diameter carbon fiber into a borosilicate glass capillary tube, OD: 1.2 mm, ID: 0.696 mm, L: 100 mm, (Hilgenberg) using negative air pressure. Carbon fiber-containing capillary tubes were then pulled on a David Kopf model 700B vertical pipette puller (David Kopf Instruments) and the protruding fiber cut to a length of 100 μm from the tip of the pipette, and sealed with a cyanoacrylate compound. CFEs were calibrated by perfusing the electrode in the recording chamber with ACSF containing 10 μM DAHCl (Sigma) and observing the maximum resultant current (nA) to produce a “current to DA concentration” conversion factor. CFEs were backfilled with 3 M KCl. Stimulating electrodes were pulled on a Sutter P-1000 Flaming/Brown micropipette puller (Sutter Instrument) using borosilicate glass capillary tubes, OD: 1.5 mm, ID: 0.86 mm, L: 100 mm, (Sutter Instrument) and the tips were broken to yield a 50 μm diameter opening. Stimulating electrodes were backfilled with ACSF.

### Data Analysis

Changes in current amplitudes following stimulation relative to the currents 100 ms prior to stimulation were converted to relative DA concentrations using a conversion factor determined by calibrating each CFE to ACSF containing 10 μM DA⋅HCl (Sigma). The maximum concentration value observed post-stimulation was extracted from each epoch, plotted over time, and normalized to the average baseline recordings. Statistical comparisons were made using the maximum concentration value recorded for each experiment post-methamphetamine application. Comparisons were also made of the maximum percent increase reached over baseline.

Data were analyzed using a curve-fitting model in the Demon Voltammetry software that incorporates Michaelis-Menten kinetics to discern contributions of DA release and reuptake according to the following equation (Wightman et al., 1988; Wightman and Zimmerman, 1990; Yorgason et al., 2011):

$$\frac{d\left[\text{DA}\right]}{dt}=\frac{f\left[\text{DA}\right]\text{p}-{\text{V}}_{\text{max}}}{\left({\text{K}}_{\text{m}}/\left[\text{DA}\right]\right)+1}$$

Changes in the extracellular DA concentration [DA] were modeled as DA release in competition with DA reuptake (Wightman et al., 1988; Wightman and Zimmerman, 1990). The DA release per pulse, [DA]p, represents the concentration of DA released evoked by an individual electrical stimulation pulse p for a train of stimuli given at frequency *f*. The Michaelis-Menten constant Vmax represents the maximal rate of DA uptake resulting from DAT activity and correlates with the amount of DAT present. Km represents the apparent affinity of DA for DAT and is used as an approximation of the degree of DAT inhibition observed (Yorgason et al., 2011). In short trains of successive stimuli, the DA released with each subsequent pulse may vary from short-term release plasticity (Sulzer et al., 2016). We therefore refer to the total released DA ([DA]r) to represent the sum of the individual [DA]p for each pulse within a phasic-like stimulus train. Total vesicular DA release was, therefore, calculated as $\left[\text{DA}\right]\text{r}=\int_{p=1}^{n}\left[\text{DA}\right]\text{p}$, where “*n*” = the number of stimulus pulses per train (10 pulses for all experiments, unless otherwise indicated). For baseline recordings, models used a Km value of 160 nM in accordance with previous studies on the affinity of DA for DAT in rodent striatum (Wu et al., 2001). Vmax was measured at baseline and kept constant in models for the duration of experiments. The apparent Km was adjusted to the best fit for changes in DA signal decay exhibited upon methamphetamine application, in addition to any potential effects on DA uptake rates by other agents applied to the brain slices. Although methamphetamines may affect the trafficking and surface expression of DAT, it remains difficult to dissociate whether differences in DA release are due to changes in Vmax from or Km, as reported by other studies (Ramsson et al., 2011). Additionally, previous voltammetric analysis of the effects of amphetamine on brain slices did not reveal a change in Vmax (Jones et al., 1999). Nonetheless, changes in Vmax caused by methamphetamine cannot be ruled out in the current study and, thus, represents a caveat to the analysis presented herein.

### SELENOP1 Protein Purification

SELENOP1 protein, including mutants, was purified from WT C57/BL6 mouse serum with an antibody affinity column using a previously described protocol (Kurokawa et al., 2014). Monoclonal SELENOP1 antibody (9S4, RRID: AB_2617215) was coupled to AminoLink Plus Coupling Resin (Pierce) and applied to a 10 mL serological pipette. Serum was first diluted 1:2 in chilled PBS and centrifuged at 14,000 *g* for 10 min at 4°C and the supernatant containing protein collected. The supernatant was run through the column and followed by a brief wash with PBS. A wash of 1 M NaCl was then applied to the column, followed by PBS. A 50 mM glycine pH 2.5 was then run through the column to elute SELENOP1 from its bound state, and the eluate collected in 1 mL fractions in tubes containing 1 M Tris pH 8.0. Fractions were tested for protein content by adding 5 μL of eluate to 10 μL drops of Bradford Assay Reagent. After all fractions were collected, the column was rinsed with PBS until wash out reached a pH of at least 7.4. The fractions from each elution that contained the highest protein concentration were concentrated to 1 mL of stock protein of 3.6 μM using a Vivaspin Centrifugal Concentrator (Sartorius). SELENOP1 mutations were previously described in Kurokawa et al. (2014). The full-length all-Cys mutant is a full-length SELENOP1 peptide with all Sec residues mutated to Cys residues. The N-terminal fragment mutant is an all-Cys SELENOP1 N-terminal peptide lacking the C-terminal region. The Δ234-237 mutant is a full-length all-Cys SELENOP1 peptide with an essential region of the ApoER2 binding domain deleted. Thus, it is unable to bind ApoER2 as previously demonstrated (Kurokawa et al., 2014).

### Statistical Analysis

Statistical comparisons were made using the peak concentration signal recorded post-methamphetamine application. For each type of experiment, an “n” specifies data of a single brain slice taken from one animal. For each animal used, only one slice was used for a given type of experiment, and additional slices from the same animal were used for different types of experiments when possible. This exploratory study was not preregistered. Analysis of DA measurements and model fitting from recorded data were performed blind to genotype and experimental conditions, although data recording was not. Animals were not randomized, and included 22 WT and 22 KO mice. Data was not included in analysis if the baseline DA responses were less than 6 nA for evoked DA release or varied by more than 10% during baseline recordings of non-stimulated DA changes. One-way ANOVA with Tukey’s multiple comparisons test used for between-subject group comparisons between multiple groups with a single variable, and two-way ANOVA with Dunnett’s multiple comparisons test used for between-subject comparisons with multiple groups and/or more than one variable. Otherwise, unpaired *t*-test was used to compare sets of two groups. The following criteria were used for significance: at *p* < 0.05 (^∗^), *p* < 0.01 (^∗∗^), *p* < 0.001 (^∗∗∗^), and *p <* 0.0001 (^****^). All statistical analysis was executed in GraphPad Prism 6 software (GraphPad Software, Inc.). All data are represented as mean ± SEM.

## Results

### DA Release Is Reduced in SELENOP1 KO Mice

Changes in extracellular DA concentration were measured in NAc slices via FSCV (Figures 1A–C). To determine if genetic deletion of SELENOP1 affects DA release we stimulated NAc slices with either single pulses, 2-pulse trains, or 10-pulse trains at 20 Hz. Sample traces are shown in Figure 1D. SELENOP1 KO slices released less DA than WT slices in response to multiple stimulation profiles (two-way ANOVA; *F*_(1,24)_ = 17.38, *p* < 0.001). Single pulse and 2-pulse stimulation similarly evoked less DA release in SELENOP1 KO slices. A 10-pulse stimulation elicited significantly greater DA release in WT than in SELENOP1 KO slices (Tukey’s; *p* < 0.05) (Figure 1E). The mean early slope, representing the presumed release portion of evoked signals was also lower in baseline measurements from SELENOP1 KO slices (unpaired *t*-test; *t*_(8)_ = 4.364, *p* < 0.005) (Supplementary Figures 1A–C). To probe for potential differences in release probability (Cragg, 2003; Condon et al., 2019), we compared the fold change in DA release in response to 2- and 10-pulse trains relative to a single pulse within each genotype. *Post hoc* analysis following two-way ANOVA did not reveal a statistically significant difference between genotypes in terms of the 2-pulse or 10-pulse response ratio. The 10-pulse to the single pulse response ratios were roughly three-fold higher for both SELENOP1 KO and WT slices. There was a significant effect of genotype on both ratios, however, with greater values detected in SELENOP1 KO mouse slices (Figure 1F).

### SELENOP1 KO Mice Exhibit Enhanced Vesicular DA Release in Response to Methamphetamine

**FIGURE 2:**
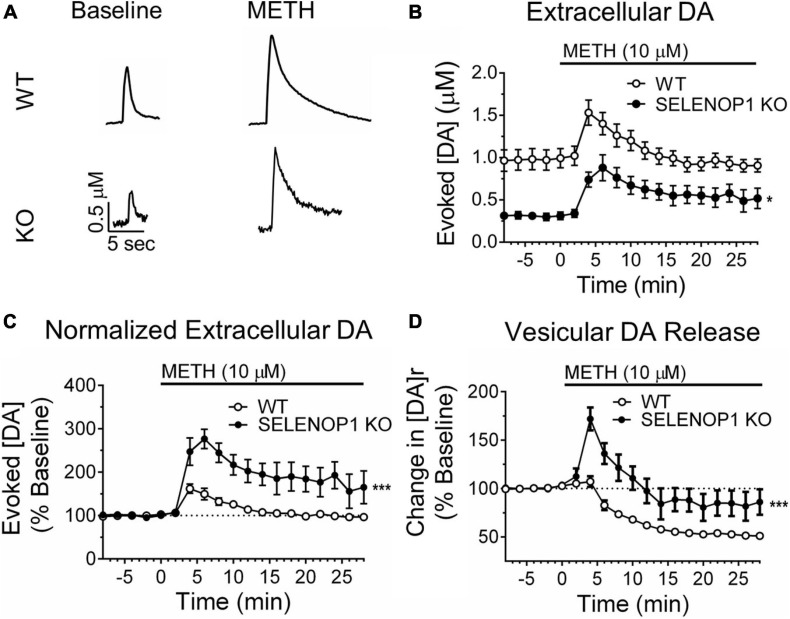
Methamphetamine enhanced vesicular DA release in SELENOP1 KO mice. **(A)** representative traces of evoked DA signals from C57 WT mice and C57 SELENOP1 KO mice before and after 10 μM methamphetamine (METH). **(B)** time course of DA release evoked in slices from WT and SELENOP1 KO mice with stimulus trains every 2 min. Peak evoked extracellular DA concentrations following exposure to METH was lower in SELENOP1 KO slices (0.9 ± 0.1 μM; *n* = 8) than in WT control slices (1.5 ± 0.1 μM; *n* = 6) (*p = 0.0127). **(C)** time course of evoked DA release represented as a percent change over baseline. Methamphetamine increased DA release in SELENOP1 KO NAc (292.9 ± 27.1%; *n* = 6) significantly more than in C57 WT NAc (163.1 ± 11.8%; *n* = 8; ***p = 0.0004) relative to baseline levels. **(D)** time course of vesicular DA release in response to methamphetamine as a percent change over baseline. Vesicular DA release is represented by the variable [DA]r, the total concentration of DA released per stimulation train. Methamphetamine induced a slight increase in [DA]r in WT mice that subsequently dropped below baseline. SELENOP1 KO mice exhibited a substantially greater increase in [DA]r upon methamphetamine application (171.9 ± 1.86%; *n* = 6) compared to WT mice (112.9 ± 3.14%; *n* = 8; ***p = 0.0001). All values reported are mean ± SEM.

We next examined whether SELENOP1 KO mice have an altered response to methamphetamine. SELENOP1 KO slices and WT slices both exhibited an immediate increase in the evoked DA response post-methamphetamine application (Figures 2A,B). Although the max post-stimulation extracellular DA concentration observed in SELENOP1 KO slices in the presence of methamphetamine was smaller compared to WT slices (*t*_(12)_ = 2.293, *p* < 0.05) (Figure 2B), the percent increase from baseline was nearly double that of WT slices (*t*_(12)_ = 4.82, *p* < 0.001) (Figure 2C). These evoked DA signals gradually decayed toward baseline levels, although decay was slower in SELENOP1 KO slices.

The curve-fitting analytical model in the Demon Voltammetry software simulates Michaelis-Menten kinetics to resolve the release and uptake components of the evoked DA signal, which are occurring simultaneously and in opposition to each other for the duration of the signal (Supplementary Figure 2A; Yorgason et al., 2011). We used this modeling system to estimate the magnitude of total vesicular DA release, [DA]r. Consistent with the observed reductions in peak extracellular DA concentration and reduction in the rising slope of baseline signals, SELENOP1 KO slices also exhibited lower [DA]r at baseline compared to WT slices (*t*_(27)_ = 4.188, *p* < 0.001) (Supplementary Figure 1D). In response to methamphetamine, SELENOP1 KO slices exhibited a robust initial increase in [DA]r that gradually decreased in amplitude toward baseline with successive stimulations (Figure 2D and Supplementary Figure 2B). In contrast, WT slices displayed only a slight increase that quickly dropped below baseline. The averaged max percent increase over baseline in [DA]r was greater in the SELENOP1 KO slices than in WT controls (*t*_(12)_ = 5.481, *p* < 0.0001). In order to observe DA efflux caused by methamphetamine, separate experiments were conducted during which changes in extracellular DA concentration were monitored in the absence of stimulation before and during methamphetamine application. There was no significant difference in the peak non-stimulated response to methamphetamine detected between WT (10.6 ± 2.3 μM) and SELENOP1 KO (6.9 ± 1.1 μM) mice (student’s *t*-test: *p* = 0.25; data not shown).

### DA Uptake Is Impaired in SELENOP1 KO Mice

We used Michaelis-Menten kinetic modeling to depict changes in DA uptake in SELENOP1 KO mice. Vmax represents the maximal rate of DA uptake when available DAT are saturated with DA. The initial decay of the evoked DA signal following DA release is primarily controlled by Vmax (Supplementary Figure 2A). Vmax was reduced in SELENOP1 KO slices, indicating slower basal DA uptake rates compared to WT slices (*t*_(44)_ = 7.021, *p* < 0.0001) (Supplementary Figure 2C).

Methamphetamine inhibition of DAT was calculated as the Michaelis-Menten constant Km, representing the apparent affinity of DA for DAT. The latter portion of the DA signal decay is taken to be largely a function of the apparent Km (Supplementary Figure 2A). Methamphetamine elicited comparable increases in apparent Km in both WT and SELENOP1 KO slices indicating similar levels of DA uptake inhibition (Supplementary Figure 2D).

Several studies have shown that gender can impact phenotypic differences in SELENOP1 KO mice that can be mitigated by selenium supplementation (Hill et al., 2003, 2004; Valentine et al., 2005; Raman et al., 2012). However, we did not find any difference between male and female mice within each genotype for the differences in DA release and reuptake kinetics reported above (Supplementary Figures 3A–I).

### SELENOP1 KO Mice Have Elevated Expression of VMAT-2 and D2R

To determine potential changes in the SELENOP1 KO mice related to changes in DA release, we measured changes in protein levels in brain lysates from WT and SELENOP1 KO mice. Expression of VMAT-2 (*t*_(5)_ = 5.007, *p* < 0.01) and dopamine D2 receptor (D2R) (*t*_(5)_ = 7.268, *p* < 0.001) were both elevated in SELENOP1 KO ventral midbrain (Supplementary Figure 4A). No changes in TH expression or DAT expression were detected in ventral midbrain (Supplementary Figure 4A). No significant difference in TH expression was observed between WT and SELENOP1 KO mice in the ventral striatum, despite observing smaller electrically evoked DA signals in SELENOP1 KO slices (Supplementary Figure 4B). VMAT-2 expression was increased in SELENOP1 KO ventral striatum (*t*_(5)_ = 3.300, *p* < 0.05), further suggesting increased vesicular packaging of DA in SELENOP1 KO mice (Supplementary Figure 4B). In order to preliminarily probe for changes in the amount of vesicular DA packaging per DA terminal we compared the expression of VMAT-2 to DAT for each subject. The ratio of VMAT-2 expression to DAT expression was significantly increased in SELENOP1 KO ventral striatum (*t*_(4)_ = 3.248, *p* < 0.05; Supplementary Figures 4C,D).

### SELENOP1 Protein Can Prevent the Methamphetamine-Induced Increase in Vesicular DA Release

SELENOP1 deletion decreases brain selenium content (Hill et al., 2003). Decreased selenium availability could, in turn, contribute to our findings, potentially via reduced expression of other members of the selenoprotein family. Previous studies demonstrated that dietary selenium supplementation can reverse many neurological deficits of SELENOP KO mice (Hill et al., 2003; Schomburg et al., 2003; Nakayama et al., 2007). To test whether reduced selenium availability caused or contributed to our results, we supplemented the drinking water of SELENOP1 KO mice with selenium (1 mg/mL) immediately post-weaning to partially restore brain selenium (Hill et al., 2003; Schomburg et al., 2003; Nakayama et al., 2007). Selenium supplementation in SELENOP1 KO mice did not significantly alter baseline DA release or the peak methamphetamine response relative to non-supplemented SELENOP1 KO mice (Supplementary Figures 5A–C). Moreover, selenium supplementation did not restore baseline evoked DA signals in SELENOP1 KO NAc, nor did it affect the measurement of Vmax or Km (Supplementary Figures 5D,E). These findings argue against the possibility that the increased [DA]r and other changes in the KO mice relative to WT animals are due to an overall reduction in brain selenium levels. However, restored selenium levels did appear to extend the duration of increased [DA]r in KOs, most likely through restored expression of one or more selenoproteins other than SELENOP1.

**FIGURE 3:**
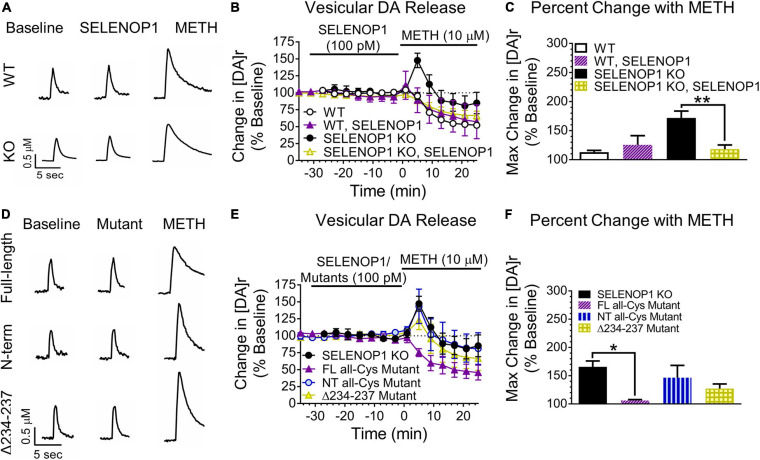
SELENOP1 protein prevented methamphetamine-induced increases in vesicular DA release via ApoER2 receptor signaling. **(A)** sample traces from WT and SELENOP1 KO slices treated with purified SELENOP1 protein (100 pM) and subsequent methamphetamine exposure. **(B,C)** SELENOP1 protein reduced the [DA]r response to methamphetamine in SELENOP1 KO mice to a level comparable to WT mice (118.2 ± 7.3%; *n* = 4; One-way ANOVA: *F*_(3,17)_ = 10.98; Tukey’s: **p = 0.0035). SELENOP1 treatment had no effect on the WT response. **(D)** sample traces of SELENOP1 KO mice when treated with various SELENOP1 mutants (100 pM) before exposure to methamphetamine. **(E,F)** pre-treatment with a full-length all-Cys SELENOP1 mutant lacking selenium (FL all-Cys Mutant) was successful in preventing the methamphetamine-induced increase in [DA]r in SELENOP1 KO mice (106.0 ± 1.7%; *n* = 3; One-way ANOVA: *F*_(3,11)_ = 4.586; Tukey’s *p = 0.0124). Treatment with an all-Cys N-terminal region SELENOP1 peptide (NT all-Cys Mutant) lacking the C-terminus, however, did not prevent the increase in [DA]r in response to methamphetamine (146.5 ± 21.7%; *n* = 3). The Δ234-237 SELENOP1 mutant that is unable to bind ApoER2 (Δ234-237 Mutant) also did not reduce the [DA]r response (127.2 ± 8.2%; *n* = 3). All values reported are mean ± SEM.

Next, we tested whether the methamphetamine-induced increase in [DA]r in SELENOP1 KO NAc slices could be prevented by pre-treatment with purified SELENOP1 protein. We applied SELENOP1 (100 pM) to brain slices via perfusion for 30 min immediately before methamphetamine application (Hollenbach et al., 2008). SELENOP1 protein by itself did not change DA release or uptake in either WT or SELENOP1 KO slices (Figure 3A). However, SELENOP1 suppressed the methamphetamine-induced increase in vesicular DA release in SELENOP1 KO slices, effectively rescuing the KO phenotype, without altering the response to methamphetamine in WT slices (Figure 3B). The change in [DA]r was significantly lower in SELENOP1-treated SELENOP1 KO slices than in non-treated SELENOP1 KO slices (*F*_(3,17)_ = 2.284, *p* < 0.001) (Figure 3C).

To determine whether the SELENOP1 was changing the methamphetamine responses by delivering selenium to NAc slices, we utilized a full-length (FL) all-Cys SELENOP1 mutant. All 10 Sec residues were changed to Cys residues in this mutant, eliminating the selenium content and preventing selenium delivery. Pre-treatment with the FL all-Cys SELENOP1 mutant to SELENOP1 KO slices resulted in a robust suppression of the methamphetamine-induced vesicular DA release, despite lacking selenium (Figures 3D–F). This demonstrates that SELENOP1 works through a selenium-independent mechanism to rescue the SELENOP1 KO phenotype.

The N-terminal domain contains several functional sites, including heparin and metal-binding regions and a redox motif. To determine if one of these properties could be responsible for the actions of SELENOP1 on slices, we utilized a mutant consisting of just the N-terminal domain fragment (NT) of the all-Cys SELENOP1 mutant. Pre-treating slices with the NT mutant resulted in an increase in [DA]r in response to methamphetamine comparable to untreated SELENOP1 KO slices (Figures 3D–F). The ineffectiveness of the NT mutant to rescue the SELENOP1 KO phenotype indicates that the SELENOP1 protein requires the C-terminal domain.

SELENOP1 binds to the apolipoprotein E receptor 2 (ApoER2) for selenium delivery. Other ApoER2 ligands such as reelin can initiate intracellular signaling (Bock and May, 2016). Previous studies have not addressed a potential role for SELENOP1 in ApoER2-mediated signaling. The ApoER2 binding site of SELENOP1 is in the C-terminal domain (Kurokawa et al., 2014). To explore the possibility that interaction of SELENOP1 with ApoER2 is a contributing factor, we used an all-Cys SELENOP1 mutant in which an essential region (residues 234–237) for ApoER2 binding is deleted, eliminating the ability of SELENOP1 to bind ApoER2 (Kurokawa et al., 2014). The mutated peptide without the ApoER2 domain (Δ234-237) did not prevent the methamphetamine-induced [DA]r increase (Figures 3D–F). One-way ANOVA revealed that a significant reduction in the [DA]r response to methamphetamine occurred only following pre-treatment with the FL all-Cys mutant (*F*_(3,11)_ = 1.128, *p* < 0.05). These data demonstrate that SELENOP1-ApoER2 interaction is required to attenuate the increased methamphetamine response in SELENOP1 KO slices.

### D2R Activity Underlies Altered Methamphetamine Response in SELENOP1 KO NAc and Rescue by Purified SELENOP1 Protein

**FIGURE 4:**
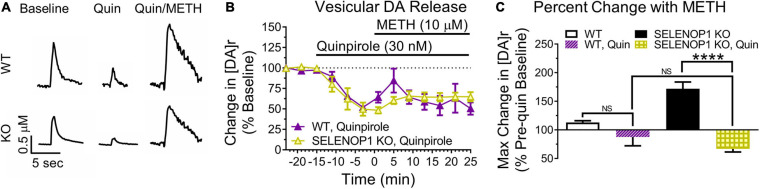
The D2R agonist quinpirole prevented methamphetamine-enhanced vesicular DA release in SELENOP1 KO NAc. **(A)** representative DA signal traces of quinpirole-enhanced D2R auto-inhibition of evoked DA release from C57 WT mice and C57 SELENOP1 KO mice, aged 3–5 months. **(B)** quinpirole (Quin; 30 nM) reduced basal DA release in WT (–54.1 ± 2.8%; *n* = 3) and SELENOP1 KO (–58.4 ± 3.9%; *n* = 3) mice similarly (*p* = 0.4). Measurements followed 15 min of quinpirole exposure (last stimulation prior to 10 μM methamphetamine (METH) application). Quinpirole also suppressed the methamphetamine-induced increase in [DA]r in SELENOP1 KO mice. WT and SELENOP1 KO mice had comparable responses to methamphetamine following quinpirole. **(C)** mean (± SEM) changes in [DA]r in response to quinpirole and methamphetamine compared to pre-quinpirole baseline levels using a two-way ANOVA. Quinpirole reduced the methamphetamine responses in SELENOP1 KO slices (66.8 ± 5.3%; *n* = 3; ****p < 0.0001). Values shown here for WT and SELENOP1 KO groups without quinpirole are the same data previously shown in Figure 3C. All values reported are mean ± SEM.

Amphetamines reportedly have an excitatory effect on DA neuron firing that is masked by D2R auto-inhibition (Shi et al., 2000). We therefore investigated if the substantial increase in [DA]r induced by methamphetamine in SELENOP1 KO mice was due to a change in presynaptic D2R. To determine whether increasing D2R activity would prevent the methamphetamine-induced [DA]r increase in SELENOP1 KO mice, we applied the selective D2R agonist quinpirole to SELENOP1 KO and WT slices for 15 min prior to and for the duration of methamphetamine exposure. Quinpirole activates presynaptic D2R to increase auto-inhibition of vesicular DA release to reduce evoked DA responses measured through FSCV. Exposure to 30 nM quinpirole for 15 min caused a similar decrease in evoked DA release in WT and SELENOP1 KO slices (Figures 4A,B). Methamphetamine increased the [DA]r in both WT and SELENOP1 KO slices following quinpirole application but did not restore the [DA]r to pre-quinpirole levels (Figure 4B). The maximum [DA]r reached in SELENOP1 KO slices as a percentage of original pre-quinpirole baseline was much smaller than the increase typically observed in SELENOP1 KO slices without quinpirole application (Figure 4C) (*F*_(3,16)_ = 2.349, *p* < 0.0001). WT and SELENOP1 KO slices treated with quinpirole exhibited comparable percent increases in [DA]r during methamphetamine application.

**FIGURE 5:**
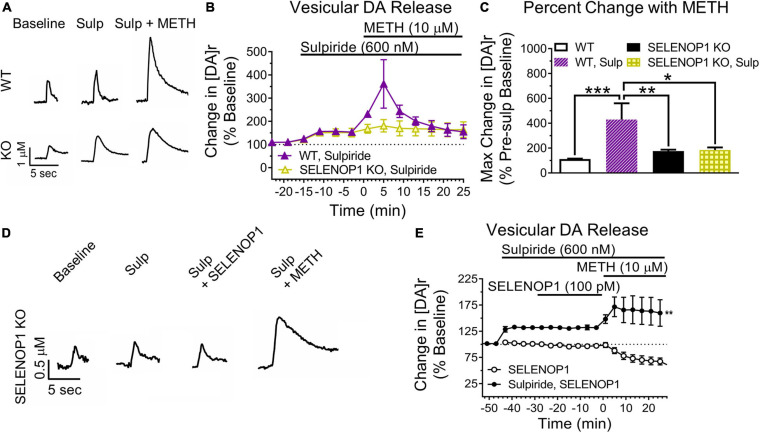
D2R antagonism unmasked elevated vesicular DA release in WT while preventing rescue in KO mice. **(A)** shown are sample traces showing sulpiride (Sulp; 600 nM) reduction of D2R auto-inhibition and subsequent responses to 10 μM methamphetamine (METH). **(B)** sulpiride increased the baseline evoked DA release from baseline values in WT and SELENOP1 KO mice similarly (148.5 ± 4.6% and 139.8 ± 6.1%, respectively; *n* = 4, 7; *p* = 0.4). **(C)** sulpiride caused a dramatic increase in [DA]r in WT mice (429.3 ± 131.4%; *n* = 3; One-way ANOVA: *F*_(3,17)_ = 10.72; Tukey’s *p = 0.0111, **p = 0.0027, ***p = 0.002), while resulting in no changes to subsequent methamphetamine responses in SELENOP1 KO mice (176.3 ± 10.4%; *n* = 6) compared to the SELENOP1 KO methamphetamine responses without sulpiride (171.9 ± 1.86%; *n* = 6). Data are expressed relative to baseline values before sulpiride application and compared to non-sulpiride methamphetamine experiment responses. Values shown here for WT and SELENOP1 KO groups without sulpiride are the same data previously shown in Figure 3C. **(D)** SELENOP1 KO mouse sample DA traces following application of sulpiride and SELENOP1 protein prior to methamphetamine. **(E)** sulpiride prevented SELENOP1 protein from suppressing the methamphetamine-induced increase in [DA]r in SELENOP1 KO mice (203.4 ± 23.9%; One-way ANOVA: *F*_(3,17)_ = *n* = 4; **p = 0.0069). All values reported are mean ± SEM.

Next, we blocked D2R auto-inhibition with the D2R antagonist sulpiride. We predicted that sulpiride would unmask methamphetamine-enhanced vesicular DA release in WT slices. Sulpiride application (600 nM) increased evoked DA release similarly in both SELENOP1 and WT slices (Figure 5A). Methamphetamine exposure post-sulpiride application dramatically increased [DA]r in slices from WT mice, eliciting a more pronounced phenotype than what was observed in non-sulpiride exposed WT and SELENOP1 KO slices (*F*_(3,16)_ = 2.445, *p* < 0.001) (Figures 5B,C). The response in sulpiride-exposed WT slices was also larger than sulpiride-exposed SELENOP1 KO slices. Sulpiride with methamphetamine did not further increase [DA]r in SELENOP1 KO slices significantly above levels observed with methamphetamine alone.

**FIGURE 6:**
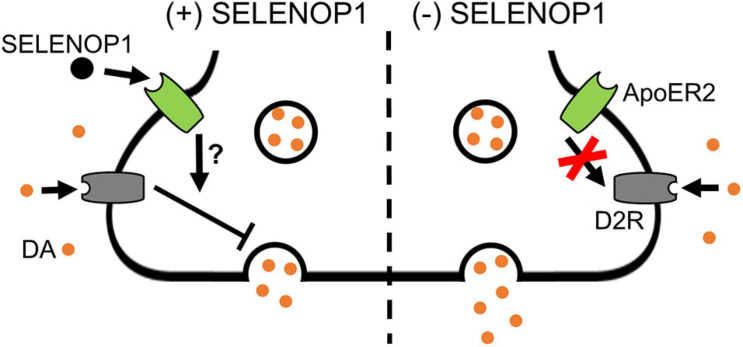
Proposed mechanism for SELENOP1 modulation of DA release. Under conditions of DA overflow beyond the synaptic cleft, such as exposure to methamphetamine, excess DA activates D2R receptors to limit DA release. SELENOP1-ApoER2 interaction partially maintains D2R activity through a mechanism that remains to be characterized (left). This mechanism could involve a direct change in D2R function, surface expression, or cross-talk between ApoER2 and D2R intracellular signaling pathways. In the absence of SELENOP1-ApoER2 interaction (right), D2R activity is deficient, allowing for augmentation of vesicular DA release. Abbreviations: ApoER2, apolipoprotein E receptor 2; DA, dopamine; D2R, dopamine receptor 2; SELENOP1, selenoprotein P.

Since sulpiride antagonism of D2R auto-inhibition unmasked a methamphetamine-induced increase in [DA]r in WT slices, we hypothesized that D2R antagonism would prevent the SELENOP1-induced rescue. To test this, we bath applied sulpiride (600 nM) prior to SELENOP1 protein, then followed by methamphetamine application. Sulpiride prevented the suppressive action of SELENOP1 protein, resulting in roughly a doubling of [DA]r over baseline once methamphetamine was added when compared to just SELENOP1-applied SELENOP1 KO slices (*t*_(6)_ = 4.022, *p* < 0.001) (Figures 5D,E). These results indicate the ability of SELENOP1 protein to directly reverse the SELENOP1 KO phenotype through an increase in D2R auto-inhibition (Figure 6).

## Discussion

We report decreased mesolimbic DA transmission, but increased vesicular DA exocytosis in response to methamphetamine, in SELENOP1 KO mice. In WT mice, a similar methamphetamine-induced increase in vesicular DA release was unmasked by blocking D2R receptor auto-inhibition. Furthermore, SELENOP1 modulated DA transmission through an ApoER2-dependent mechanism not involving selenium transport.

Electrically evoked DA signals were significantly reduced in slices from SELENOP1 KO mouse NAc compared to slices from WT mice, as shown by decreased peak extracellular DA concentration as well as a decrease in the early rising slope. These measurements agree with a decrease in total DA release, [DA]r, derived from the kinetic model. The difference between genotypes in the ratios of 2- and 10-pulse stimulation relative to single pulse (Figures 1D,E) may indicate a different release probability (Cragg, 2003; Condon et al., 2019). Following methamphetamine, the extracellular DA levels and DA release for KO slices were similar to pre-methamphetamine levels in WT slices (Figure 2C). This could indicate an overall reduction in synapse number or vesicles per terminal. A reduction in DA terminals would likely correlate with a reduction in DA terminal proteins such as DAT or DA vesicular proteins such as VMAT. However, the vesicular protein VMAT-2 expression was increased in the SELENOP1 KO ventral striatum, while expression of the presynaptic terminal protein DAT was unchanged, findings that are not consistent with a loss of dopaminergic terminals. Furthermore, our observation that the ratio of VMAT-2/DAT expression was increased in SELENOP1 KO ventral striatum suggests an increase rather than reduction in DA vesicles per terminal in the SELENOP1 KO NAc. Increased vesicles could be a consequence of decreased excitatory release that results in a build-up of releasable vesicles. Interestingly, the amplitude of DA release in response to multi-pulse stimulation increased over the response to single-pulse stimulation to a greater degree in SELENOP1 KO slices than in WT slices, suggesting a greater increase in vesicular release probability. Thus, SELENOP1 KOs may have a larger ratio of DA reserve vesicles to readily releasable vesicles in the NAc compared to WT mice. Basal DA uptake rates were reduced in SELENOP1 KO slices, which typically indicates lower DAT expression. Western blot analysis did not detect any change in DA expression in SELENOP1 KO striatum, however, which suggests that the functionality of DAT may be impaired in the SELENOP1 KO NAc under baseline conditions.

Although amphetamines are thought to primarily increase extracellular DA levels via reuptake blockade and non-vesicular release (Sulzer and Rayport, 1990; Sulzer et al., 1992; Seiden et al., 1993), some studies have suggested that amphetamines can increase vesicular release of DA (Covey et al., 2013, 2016; Daberkow et al., 2013). Covey et al. suggested that amphetamines up-regulate the readily releasable pool in ventral striatum to increase vesicular release (Covey et al., 2013). In this scenario, methamphetamine would mobilize DA to the readily releasable pool to increase the evoked DA signal and [DA]r. Thus, if a greater portion of DA is stored within the reserve pool in SELENOP1 KO mice and methamphetamine works by mobilizing this DA for release, then the mobilization of this pool of DA may contribute to the greater increase in DA release over baseline observed in SELENOP1 KO slices when methamphetamine is added. Our findings demonstrate a previously unreported function of SELENOP1 independent of selenium transport and other known properties. Supplementing SELENOP1 KO mice with selenium via drinking water showed the same peak response to methamphetamine as non-supplemented mice. Selenium supplementation did seem to mitigate the decay in vesicular DA release over time following the spike at the beginning of methamphetamine exposure, showing some effect, but it did not change the early kinetics. Selenium supplementation restores brain selenium levels and reverses selenium-related impairments (Hill et al., 2003; Schomburg et al., 2003; Nakayama et al., 2007). It is possible that the amount of selenium ingested via drinking water may have varied between each mouse. However, the variability of the data collected from selenium-supplemented mice was similar to that taken from non-supplemented mice with equal samples sizes (Supplementary Figure 5). Therefore, the altered methamphetamine response in the SELENOP1 KO mice does not appear to be due to reduced brain selenium levels. Neurodevelopmental changes in the DA system of SELENOP1 KO mice are possible as the SELENOP1 receptor ApoER2 facilitates DA neuronal migration during development (Sharaf et al., 2013, 2015). However, the observation that short-term application of SELENOP1 could restore the methamphetamine response to WT levels argues against major developmental impairments. Moreover, the full-length all-Cys SELENOP1 mutant lacking selenium was as effective as the non-mutated full-length SELENOP1 at restoring the methamphetamine response. The truncated N-terminal fragment was ineffective, however, ruling out several functions of the N-terminal domain. These include the antioxidant activity of the thioredoxin-like redox motif, the binding of heparin glycoproteins, and metal binding properties (Burk and Hill, 2015). Thus the C-terminal SELENOP1 domain, which includes the ApoER2 binding site (Kurokawa et al., 2014), is necessary for the observed changes in DA release. The Δ234-237 SELENOP1 mutant, with a specific deletion of the ApoER2-binding domain of SELENOP1, was also ineffective. This indicates that the interaction of SELENOP1 with ApoER2 is necessary to restore the suppressive response to methamphetamine. SELENOP1 co-localized with DAT in postmortem human brain, indicating the presence of SELENOP1 at DA terminals (Bellinger et al., 2012). These results, taken together, provide strong evidence for SELENOP1-mediated signaling though ApoER2.

Previous studies showed that SELENOP1 binds to ApoER2 in order to mediate selenium transport across membranes (Burk et al., 2007, 2014; Olson et al., 2007). ApoER2 has a separate role in conjuction with the very-low-density-lipoprotein receptor (VLDLR) in mediating Reelin signaling (Reddy et al., 2011). Our results suggest that an additional role for SELENOP1-ApoER2 interaction is to induce a possible signal cascade to modulate DA release. ApoER2 interacts with different scaffolds and adaptor proteins, such as Dab1, which promotes ApoER2 surface expression, while ligands such as ApoE can promote ApoER2 internalization (Cuitino et al., 2005). Interestingly, the adaptor protein CIN85 binds to Dab1 to potentially mediate internalization of various membrane receptors, including D2R (Shimokawa et al., 2010; Fuchigami et al., 2013). This suggests a possible mechanism for which ApoER2 may be able to influence D2R surface expression. ApoER2 is also known to associate with the N-methyl-D-aspartate receptor (NMDAR). NMDAR activation on active pre-synaptic striatal DA terminals promotes DA release in a Ca^2+^-dependent manner (Wang, 1991). Therefore, internalization of DA terminal-resident NMDARs, post-synaptic to regulatory glutamatergic inputs, via ApoER2 activation is another possible mechanism underlying the SELENOP1-dependent limitation of DA release.

Methamphetamine is thought to primarily increase extracellular DA levels via reuptake blockade and non-vesicular release (Sulzer and Rayport, 1990; Sulzer et al., 1992; Seiden et al., 1993). However, studies have suggested that amphetamines can increase vesicular release of DA (Covey et al., 2013, 2016; Daberkow et al., 2013). Covey et al. (2013) suggested that amphetamines up-regulate the readily releasable pool in ventral striatum to increase vesicular release. In this scenario, methamphetamine would mobilize DA to the readily releasable pool to increase the evoked DA signal and [DA]r.

We observed increased vesicular release not only in SELENOP1 KO mice, but also in WT animals in the presence of a D2R antagonist. Pre-application of the D2R antagonist sulpiride revealed a methamphetamine-induced increase in vesicular release in WT slices independent of DAT inhibition. Shi et al. (2000) reported that amphetamine causes an excitation in VTA DA neurons, which is masked by D2R activation via amphetamine-elevated DA concentrations. Thus, D2R autoreceptors may prevent the observation of increased vesicular release. It is worth noting that, in our experiments, pre-application of sulpiride did not potentiate the response to methamphetamine in SELENOP1 KO slices to as great of an extent as in WT slices. It is possible that this is because D2R autoreceptors are already unable to limit vesicular DA release in the SELENOP1 KO NAc in the presence of methamphetamine. Further interrogation of this relationship would benefit from including dose-response curves for these different pharmacological treatments and, thus, represents a limitation of the current study.

The prevention of a methamphetamine-induced increase in vesicular release in SELENOP1 KO phenotype by exogenous SELENOP1 likely involves D2R activity. The D2R agonist quinpirole prevented the large methamphetamine-induced increase of [DA]r in SELENOP1 KO slices. This finding implies reduced D2R activity in the SELENOP1 KO NAc, which is accentuated in the context of methamphetamine exposure. Sulpiride prevented SELENOP1 protein from increasing stimulated DA release in SELENOP1 KO slices, suggesting activation of a signaling pathway that restores D2R activity and limits increases in vesicular DA release. This pathway appears to involve SELENOP1-ApoER2 interaction, as a mutation to the ApoER2-binding domain of SELENOP1 prevented the rescue of the KO phenotype. Taken together, these results suggest that SELENOP1-ApoER2 binding normally promotes D2R function, likely auto-inhibitory, which masks the methamphetamine enhancement of vesicular DA release. In the absence of SELENOP1, D2R activity may be decreased, allowing for the large increases in [DA]r we observed. This proposed mechanism is summarized in Figure 6. Further investigation is needed to determine the pathways through which ApoER2 regulates D2R. Among the possibilities are (1) changes in D2R surface expression, (2) changes in D2R functionality, and (3) cross-talk between ApoER2 and D2R intracellular signaling pathways. Interestingly, mice with heterozygous genetic deletion of the ApoER2 ligand Reelin exhibit region-specific alterations in D2R expression, with both increases and decreases reported occurring in the striatum (Varela et al., 2015).

The results described herein directly implicate SELENOP1 as an important regulator of DA transmission, a role not previously reported. In contrast to several studies that have reported elevated DA turnover in rats in response to dietary selenium restriction (Castano et al., 1997; Rasekh et al., 1997; Romero-Ramos et al., 2000), we demonstrate decreased basal DA release in SELENOP1 KO mouse striatal slices. The previous reports are not necessarily in conflict with our findings, however, as these studies reported DA and DA metabolites measured over longer periods of time (hours and days) compared our study (minutes in duration with sub-second temporal resolution). Elucidating this relationship sheds further light on the protective actions of selenium against methamphetamine-induced neurotoxicity (Imam et al., 1999; Kim et al., 1999; Barayuga et al., 2013) by demonstrating the ability SELENOP1 to limit extracellular DA transmission. This can potentially limit damage to dopaminergic terminals caused by excessive DA auto-oxidation that result from excessive dopaminergic activity, such as that caused by methamphetamine (Cadet and Brannock, 1998). The current study also improves our understanding of the methamphetamine mechanism of action as it provides corroborating evidence that methamphetamine increases vesicular DA release, a phenomenon reported for amphetamine in several previous studies (Covey et al., 2013, 2016; Daberkow et al., 2013). Amphetamine-induced elevations in extracellular DA in rodent NAc slices are dependent on DAT (Siciliano et al., 2014). However, the measured increases in [DA]r observed in this study are likely independent of DAT inhibition, as methamphetamine-influenced reuptake kinetics in SELENOP1 KO slices were comparable to WT slices. These data may be relevant to addiction since DA release events are critical in reward-based learning and drug reinforcement (Stuber et al., 2005; Steinberg et al., 2014), and the NAc shell is thought to play a more significant role in addiction compared to the NAc core (Ikemoto, 2007).

We previously reported the association of SELENOP1 with lesions of both Alzheimer’s disease (Bellinger et al., 2008) and Parkinson’s disease (Bellinger et al., 2012), suggesting a role in neurodegeneration. Given that dopaminergic terminals are particularly vulnerable to damage such as that from DA auto-oxidation, the demonstrated ability of SELENOP1 to limit DA release raises the possibility of a neuroprotective role in neurodegenerative diseases and aging (Kumar et al., 2012). Importantly, ApoER2 is also a receptor for ApoE, for which the e4 polymorphism is the most prominent genetic risk factor for Alzheimer’s disease (Zhao et al., 2018). One possibility is that ApoE limits the protective influence of SELENOP1 by competing for ApoER2 binding or reducing ApoER2 surface expression (Chen et al., 2010). In addition to Alzheimer’s disease, ApoE has been implicated in other diseases such as parkinsonism (Jellinger, 2018) and HIV-related dementia (Olivier et al., 2018), further highlighting SELENOP1-ApoER2 interaction as an area of interest in neurodegeneration research.

This study demonstrates dopaminergic regulation by SELENOP1. We show that genetic deletion of SELENOP1 results in increased DA vesicular release in response to methamphetamine, and that addition of exogenous SELENOP1 prevents this increase. The direct actions of SELENOP1 involve (1) binding to ApoER2 and (2) D2R activity. Furthermore, we demonstrate that D2R receptor auto-inhibition masks an increase in vesicular DA release in WT mice. Our findings show that SELENOP1 can act to modulate neurotransmission through a mechanism other than selenium delivery, further expanding its role in the brain.

## Data Availability Statement

The original contributions presented in the study are included in the article/Supplementary Material, further inquiries can be directed to the corresponding author.

## Ethics Statement

The animal study was reviewed and approved by the University of Hawai‘i at Mânoa Institutional Animal Care and Use Committee (UH Manoa IACUC).

## Author Contributions

FB, DT, JY, SS, SK, and MA designed the research. DT, CM, and AH performed the research. DT, JY, CM, and FB analyzed the data. DT and FB wrote the manuscript. All authors contributed to the article and approved the submitted version.

## Conflict of Interest

The authors declare that the research was conducted in the absence of any commercial or financial relationships that could be construed as a potential conflict of interest.
