# Supplementary material for: Selenoprotein P Modulates Methamphetamine Enhancement of Vesicular Dopamine Release in Mouse Nucleus Accumbens Via Dopamine D2 Receptors
Source: Front Neurosci. 2021 Apr 13;15:631825. doi: 10.3389/fnins.2021.631825 (PMC8076559; doi:10.3389/fnins.2021.631825)
Supplement: Supplementary file 1 [file Table_1.docx]

Supplementary Material - Selenoprotein P Modulates Methamphetamine Enhancement of Vesicular Dopamine Release in Mouse Nucleus Accumbens via Dopamine D2 Receptors

**SUPPLEMENTARY METHODS**

*Gel electrophoresis and western blotting.* Dissected brain parts were pulverized using the CryoGrinder kit (OPS Diagnostics). The ceramic mortar was placed on powdered dry ice along with a ceramic pestle and metal scooper, covered and allowed to equilibrate for 10 minutes. Next, the individual brain part was placed in the mortar and ground into powder using the pestle attached to a Black and Decker drill. One half of the powder was added to a tube containing 300µL CelLytic MT Mammalian Tissue Lysis/ Extraction Reagent (Sigma) containing 1:100 protease inhibitor cocktail (Sigma) while the other half was placed in an empty tube for future studies. Lysis buffer with suspended tissue was sonicated with 20 one-second pulses at 5 Hz, separated by one second each, using a Fisher Sonic Dismembrator Model 100 (Fisher Scientific). Samples were then centrifuged at 14,000xg for 10 minutes at 4°C. The supernatant was collected and stored at -80°C for western blotting.

Tissue lysate samples containing 40 µg of protein were separated on 4-20% gradient polyacrylamide TGX gels (BIO-RAD) via electrophoresis and transferred to 0.45 µm pore size Immobilon-FL polyvinylidene difluoride membranes (Millipore). Membranes were incubated in PBS-based blocking buffer (LICOR) for 1 hour and then probed with primary antibodies for 1.5 hours, followed by washing with PBS containing 0.01% Tween 20 (Fisher Scientific) (PBS-T). Blots were incubated with infrared fluorophore-bound secondary antibodies in the dark, washed again with PBS-T, and analyzed using the Odyssey Imaging System (LI-COR Biosciences).

*Antibodies.* Primary antibodies used for western blotting were: rabbit anti-TH (Cell Signaling Technology Cat# 2792S, RRID:AB_10691683); rabbit anti-DAT (Millipore Cat# AB2231, RRID: AB_1586991), rabbit anti-VMAT-2 (Millipore Cat# AB1598P, RRID:AB_2285927); rabbit anti-D2R (Millipore Cat# AB5084P, RRID:AB_2094980), monoclonal mouse anti-αTubulin (Thermo Fisher Scientific Cat# 62204, RRID:AB_1965960). Secondary antibodies used for western blotting were LI-COR IRDye highly cross-adsorbed antibodies optimized for use with the Odyssey Imaging System with emission wavelengths of either 696 or 795 nm and directed against the primary antibody species.

**
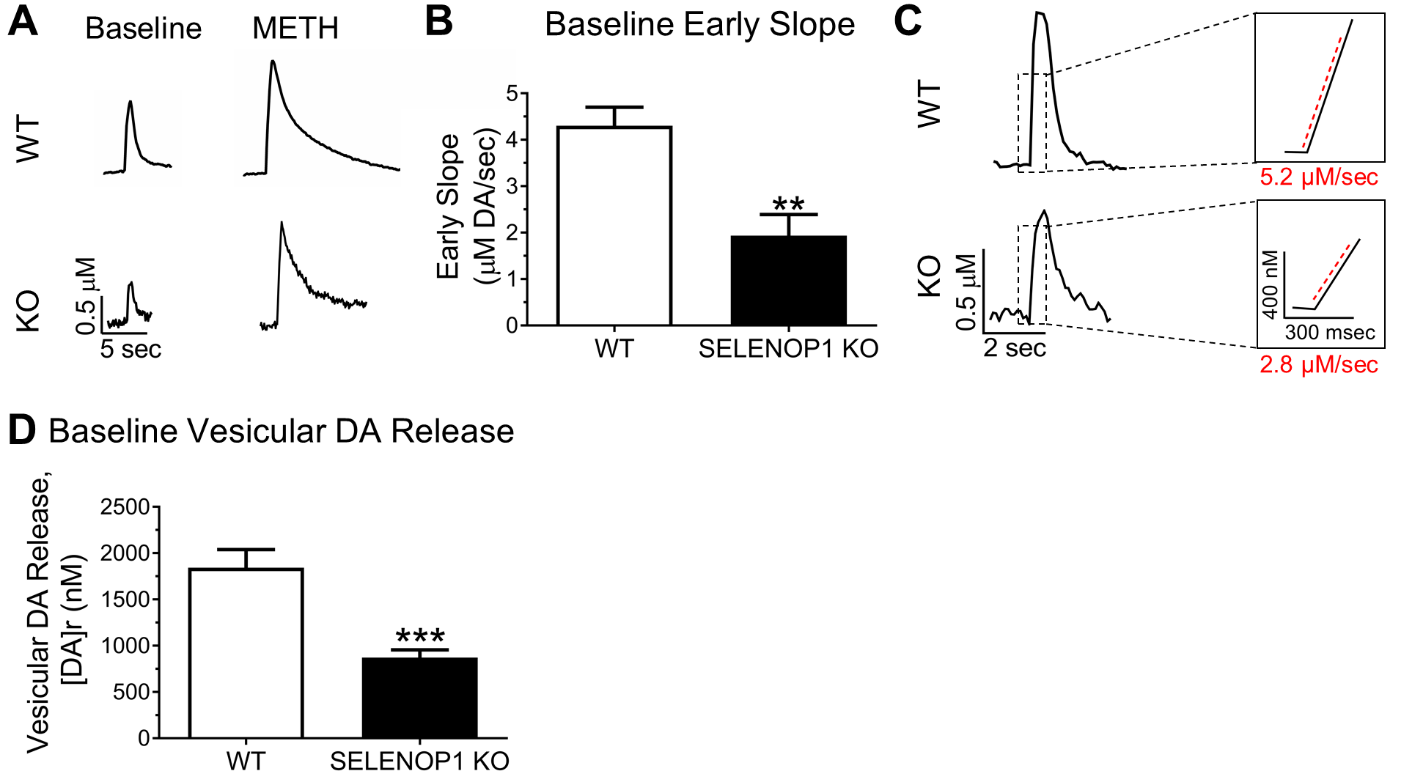
**

**Supplementary Figure 1** Baseline early slope, extracellular DA with METH and baseline vesicular DA release in SELENOP1 KO mice. (**A**) Sample baseline traces from WT and SELENOP1 KO slices (also found in Figure 2). (**B**) Mean (± SEM) early rising slope derived from the rising phase of the DA release signal. Early slope is reduced in SELENOP1 KO mice (1.9 ± 0.5 µM/sec; n = 6) compared to WT controls (4.3 ± 0.4 µM/sec; n = 4) at baseline (***p* = 0.0042). (**C**): Magnification of rising slope of baseline traces from WT and SELENOP1 KO slices. (**D**) Total DA released, [DA]r, was also reduced (873 ± 82 nM; n = 12) compared to controls (1842 ± 197 nM; n = 16) at baseline (****p* = 0.0003). All values reported are mean ± S.E.M.


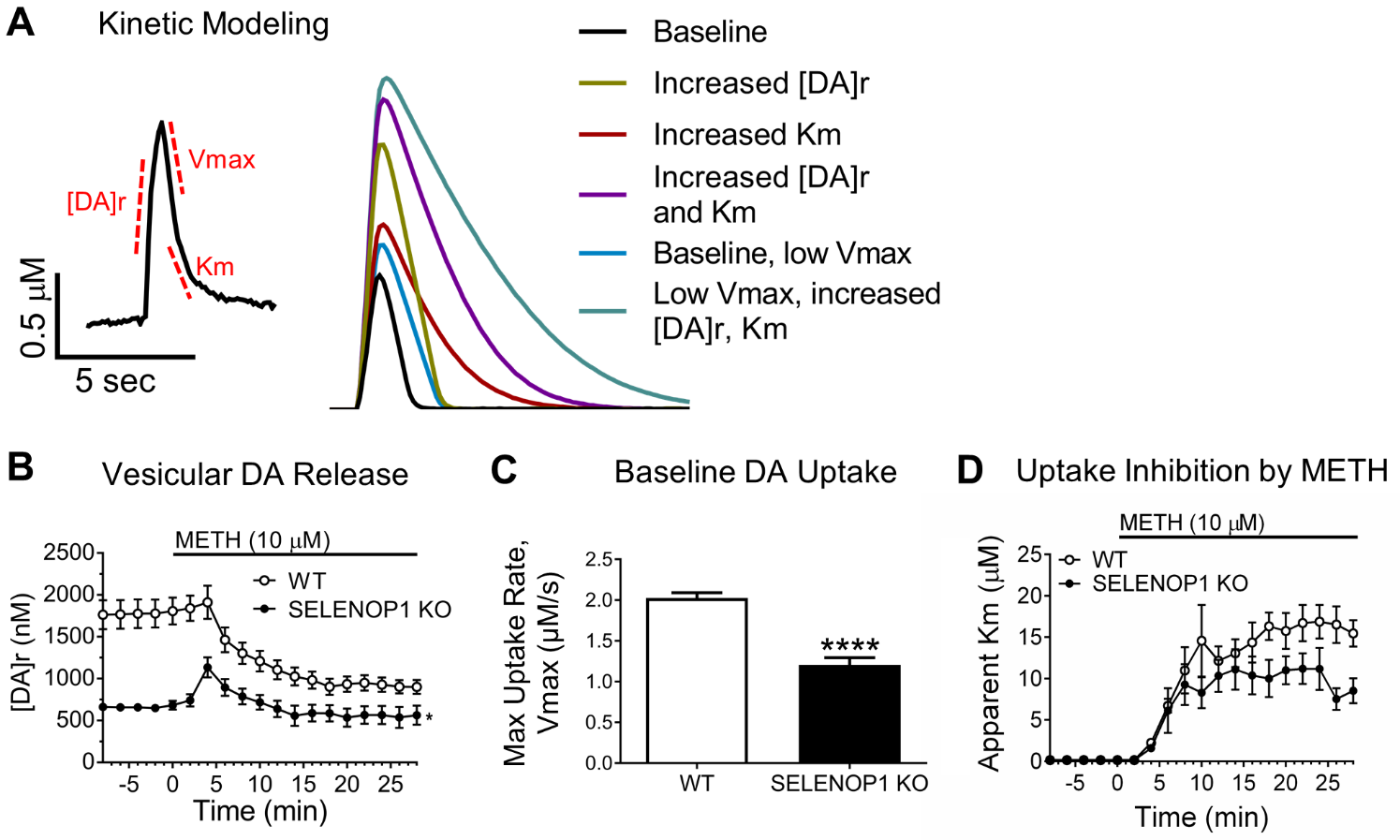


**Supplementary Figure 2** Description of kinetic modeling, baseline DA uptake and methamphetamine-induced uptake inhibition. (**A**) Data modeled from intial baseline measurements (left) to show how changes in various kinetic parameters should alter reordings (right). DA uptake rates were calculated from baseline using a kinetic analysis model according to the following equation: $\frac{d\left[ \mathrm{DA} \right]}{dt} = \frac{ƒ\left[ \mathrm{DA} \right]p -V_{\max}}{\left( {K_{m}}/\left[ \mathrm{DA} \right] \right)+1}$ where $\left[ \mathrm{DA} \right]r=\sum_{p=1}^{n} \left[ \mathrm{DA} \right]p$ and ‘n’ = the number of stimulation pulses. The Michaelis-Menten constant Vmax, which represents the maximum rate of uptake via DAT, primarily regulates the initial decay of the DA signal while Km, the apparent affinity of DA for DAT, primarily influences the latter portion of the signal decay. [DA]r, the total amount of DA released, influences the height of the evoked signal peak and the slope of the release phase. *Left:* A sample trace from a WT mouse showing the influence of Vmax and Km on signal decay in the upper and lower portions, respectively. *Right:* Computer-generated traces are shown to illustrate how increases in either [DA]r (sum of DA released per stimulation pulse) or Km would alter recorded FSCV responses, as predicted by the curve-fitting model (while keeping Vmax constant). Traces as listed in the graphic: Baseline (black) – typical baseline trace pre-methamphetamine exposure; Increased [DA]r (gold) - simulated increase in [DA]r only representing vesicular release, results in a higher peak response without changes in the rate of decay; Increased Km (red) - increased Km only, to model decreased reuptake, results in a reduced rate of decay, with a small increase in peak due to a later attenuation of the rise time; Increased [DA]r and Km (purple) – shows the combined effect of increasing [DA]r and Km by the same values as in the previous two simulations; Baseline low Vmax (blue) – same parameters as initial baseline simulation, but with a lower Vmax, showing a small increase in peak, but without the changes in decay associated with increased Km; Low Vmax, Increased [DA]r, Km (Green) – [DA]r and Km are increased in equal proportions as in the fourth (purple) simulation to illustrate the effect when Vmax is smaller to begin with. (**B**) Time course of mean (± SEM) vesicular DA release in response to methamphetamine in slices from WT and SELENOP1 KO mice. Repeated measures ANOVA revealed a significant overall effect of genotype F_(1,12)_ = 18.18; *p* = 0.001. The average [DA]r during methamphetamine application was significantly higher in WT slices (1103 ± 106.5 nM) compared to SELENOP1 KO slices (658.1 ± 94.5; student’s t-test, **p* = 0.01) (**C**) Vmax calculated for WT mice and C57 SELENOP1 KO mice. Vmax was lower in SELENOP1 KO slices (1.2 ± 0.09 µM/sec; n = 27) compared to WT slices (2.0 ± 0.06 µM/sec; n = 19; *****p* < 0.0001). WT mice and SELENOP1 KO mice, aged 3-5 months were used. (**D**) Time course of apparent Km in response to 10 µM methamphetamine (METH). Km, the affinity of DA for the transporter, was set to 160 nM at baseline according to previously published data (Wu *et al.* 2001) and increased in response to methamphetamine as DA uptake was inhibited. The maximum Km reached in the presence of methamphetamine was not significantly different between groups, indicating comparable degrees of DAT inhibition (23.8 ± 3.8 µM for WT, 14.6 ± 2.2 µM for SELENOP1 KO; n = 8, 6, respectively; *p* = 0.078). Repeated measures ANOVA did not reveal any effect of genotype (F_(1,12)_ = 2.23; *p* = 0.16), nor did post-hoc analysis reveal differences at any specific timepoint. Analysis of area under the curve also failed to reveal a significant difference (WT = 145.9 ± 22.7, SELENOP1 KO = 100.9 ± 16.2; student’s t-test, *p* = 0.16). All values reported are mean ± S.E.M.


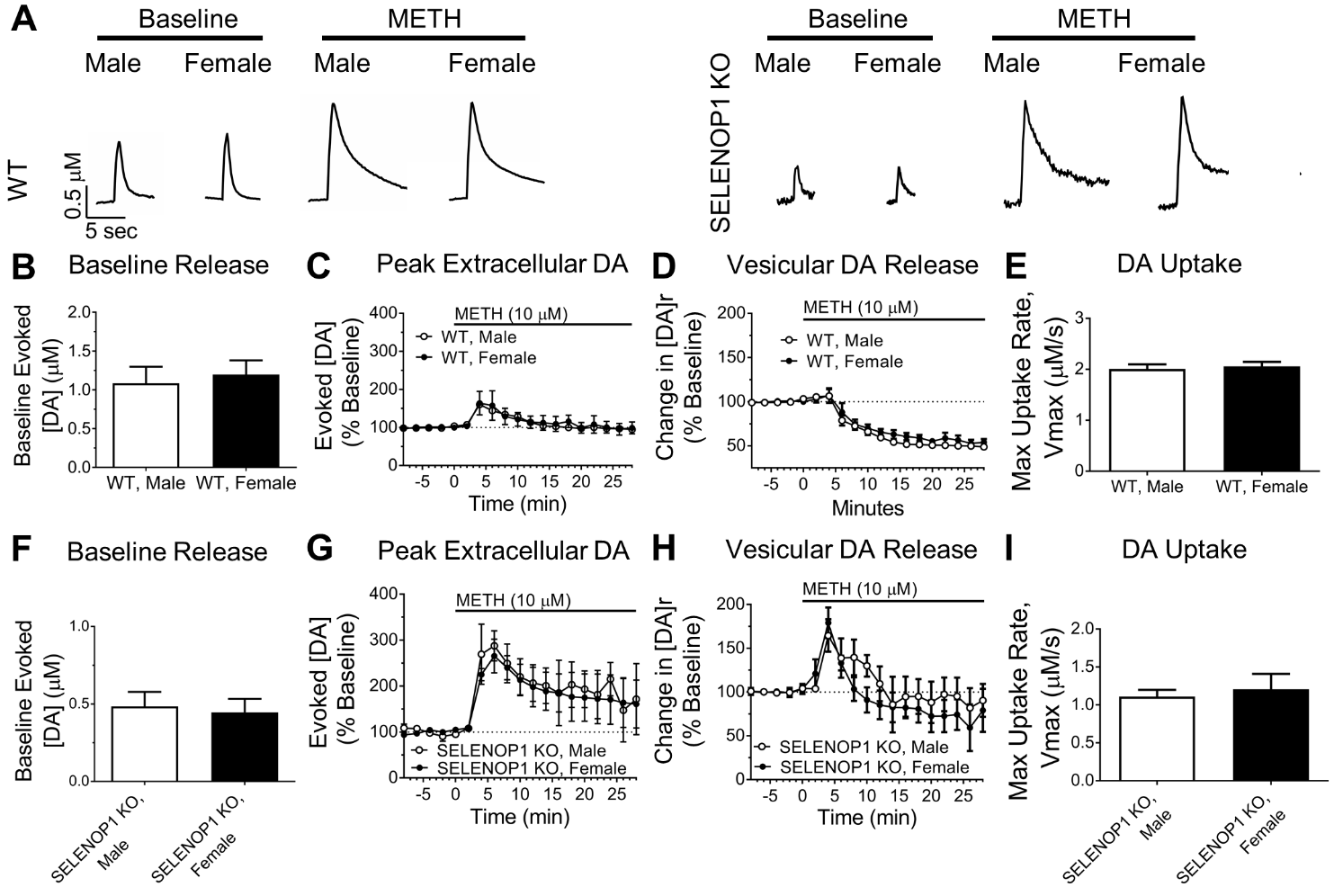


**Supplementary Figure 3** There were no sex differences in dopaminergic activity in WT or SELENOP1 KO mice. (**A**) Sample traces from male and female C57 WT mice and SELENOP1 KO mice, aged 3-5 months. Male traces are same as used in Figure 2. (**B**) Baseline evoked [DA] in NAc slices from male (1.1 ± 0.2 µM; n = 10) and female (1.2 ± 0.2 µM; n = 6) WT mice (*p* = 0.7). (**C**) Changes in extracellular [DA] in WT mice by sex in response to 10 µM methamphetamine (METH). (**D**) Changes in [DA]r in response to methamphetamine in WT mice by sex. (**E**) Basal DA uptake rates in male (2.0 ± 91.4 µM/s; n = 12) and female (2.1 ± 86.7 µM/s; n = 7) WT mice by sex (*p* = 0.7). (**F**) Baseline evoked [DA] in NAc slices from male (0.50 ± 0.1 µM; n = 9) and female (0.4 ± 0.1 µM; n = 4) SELENOP1 KO mice (*p* = 0.8). (**G**) Changes in extracellular [DA] in SELENOP1 KO mice by sex in response to 10 µM methamphetamine. (**H**) Changes in [DA]r in response to methamphetamine in SELENOP1 KO mice by sex. (**I**) Basal DA uptake rates in male (1.1 ± 89.0 µM/s; n = 18) and female (1.2 ± 203.6 µM/s; n = 9) SELENOP1 KO mice by sex (*p* = 0.6). All values reported as mean (± SEM) and comparisons between sexes made within each genotype via unpaired t-test.


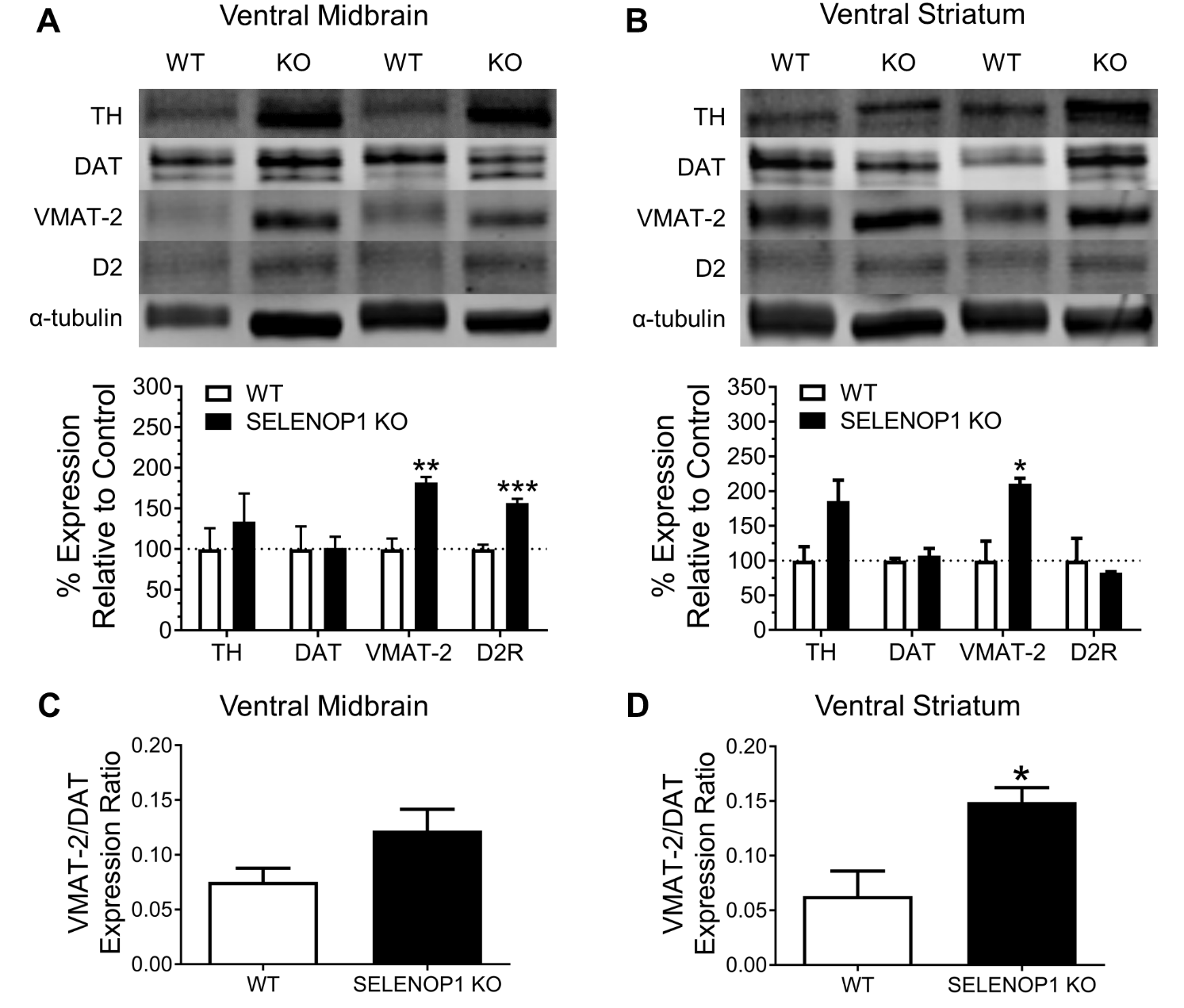


**Supplementary Figure 4** Western blot revealed SELENOP1 KO mice have increased expression of TH, VMAT-2 and D2R. (**A**) Mean (± SEM) WT and SELENOP1 KO protein expression from ventral midbrain lysates. TH: no change in expression between WT mice and SELENOP1 KO mice, both groups aged 3-5 months (n = 3, 3; *p* = 0.5). DAT: No changes (n = 4, 3; *p* = 0.9). VMAT-2: Increased expression in SELENOP1 KO mice (~182% increase over controls) (n = 4, 3; ***p* = 0.0041). D2R: Increased expression in SELENOP1 KO mice (~157% increase over controls; n = 4, 3; ****p* = 0.0008). (**B**) Mean (± SEM) WT and SELENOP1 KO protein expression from ventral striatum lysates. TH: No significant change in expression detected in SELENOP1 KO mice (~186% increase over controls; n = 4, 3; *p* = 0.055). DAT: No changes (n = 3, 3; *p* = 0.5). VMAT-2: Increased expression in SELENOP1 KO mice (~211% of control; n = 4, 3; **p* = 0.0215). D2R: no changes (n = 4, 3; *p* = 0.7). (**C**, **D**) The ratio of VMAT-2/DAT expression was significantly greater in SELENOP1 KO mouse ventral striatum (0.15 ± 0.01; n = 3) compared to WT controls (0.06 ± 0.02; n = 3; **p* = 0.0314) and no significant difference was detected in ventral midbrain (0.1 ± 0.02; n = 3 compared to controls 0.08, ± 0.01; n = 4). All values reported are mean ± S.E.M.


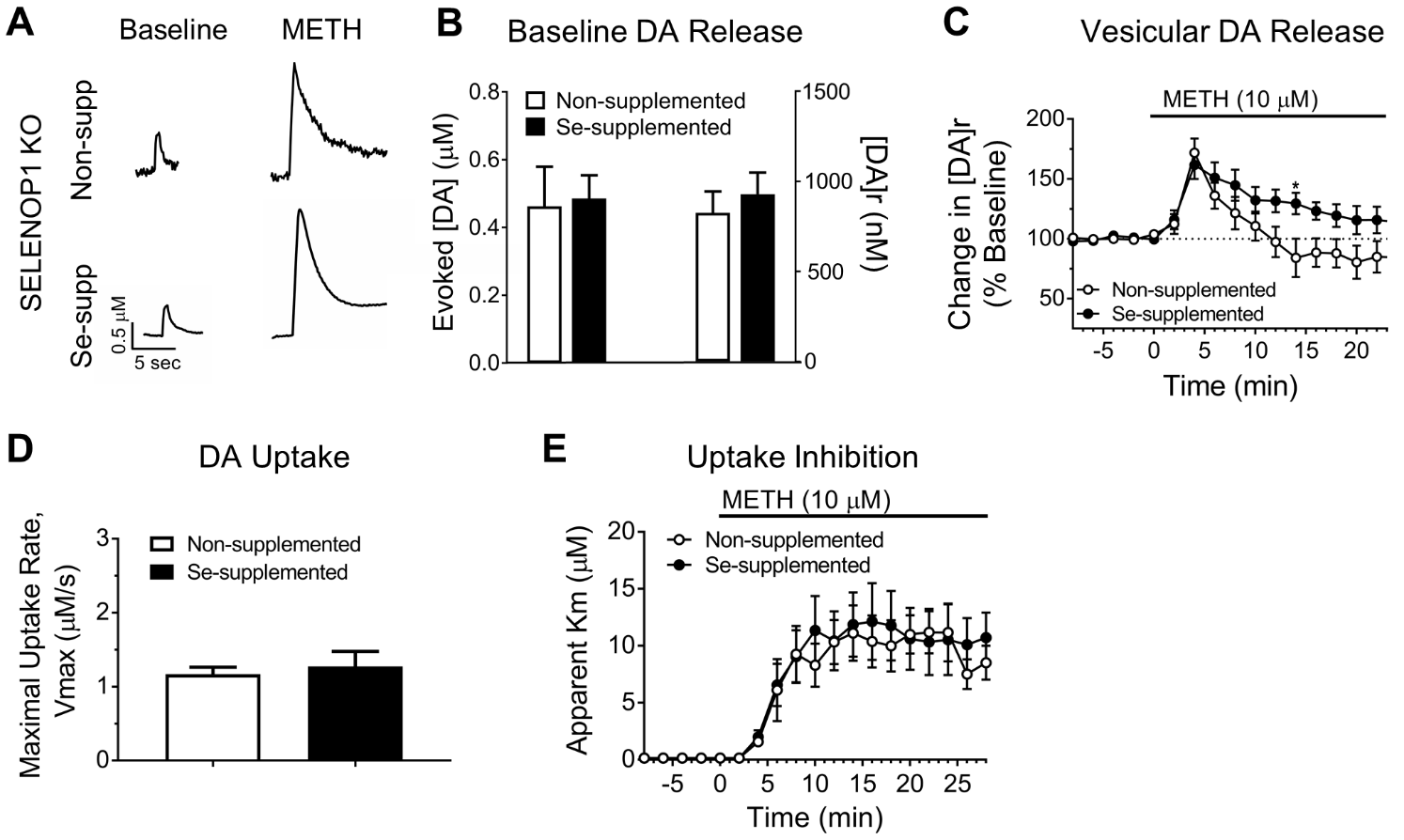


**Supplementary Figure 5** Selenium supplementation had no effect on the methamphetamine response in SELENOP1 KO mice. (**A**) Representative traces are showing 10 µM methamphetamine (METH) responses in brain slices from (aged 3-5 months) SELENOP1 KO mice with and without selenium (Se) supplementation. Se-supplemented mice were given *ad libitum* access to drinking water containing 1mg/mL Se. (**B**) Selenium supplementation had no effect on baseline DA release in SELENOP1 KO mice. (**C**) The percent increase in [DA]r in response to methamphetamine in Se- and non-supplemented mice were comparable (167.3 ± 11.8 % and 171.9 ± 11.8 %, respectively; n = 6, 6; *p* = 0.8). Repeated measures ANOVA did reveal a significant difference between Se- and non-supplemented groups at 14 minutes of methamphetamine exposure (Sidak’s multiple comparisons test, **p* = 0.04) (**D**) Selenium supplementation did not change Vmax or (**E**) methamphetamine-induced DA uptake inhibition in SELENOP1 KO mice. All values reported are mean ± S.E.M.
